# Supplementary figures and images for: L-2-hydroxyglutarate regulates centromere and heterochromatin conformation in the male germline
Source: PLoS Genet. 2025 Jul 10;21(7):e1011785. doi: 10.1371/journal.pgen.1011785 (PMC12306753; doi:10.1371/journal.pgen.1011785)

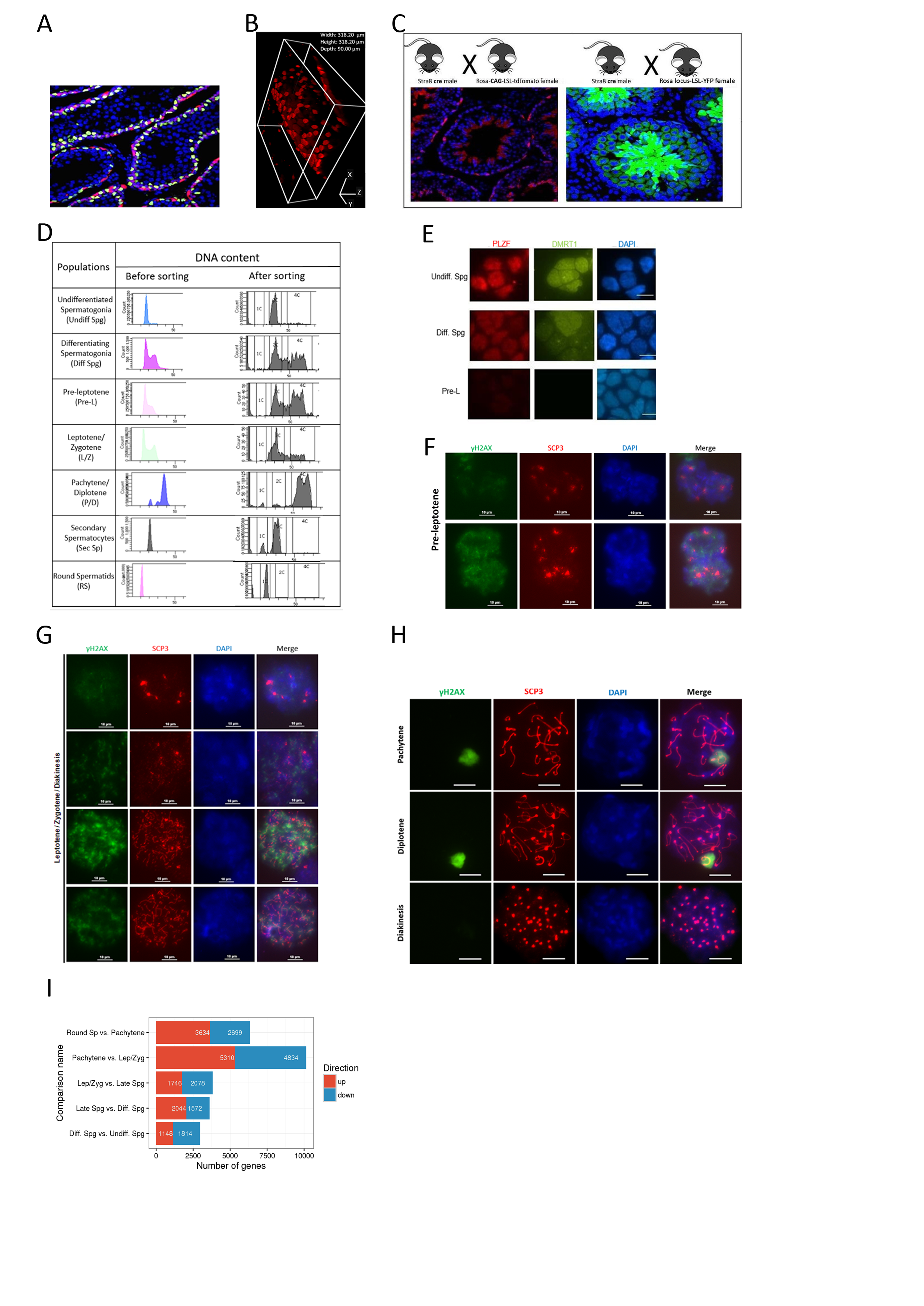

Supplement: S1 Fig — A. tdTomato fluorescence (red) and immunostaining for Sox9 (green), demonstrating germ cells specificity of tdTomato expression, frozen section of Stra8-Tom mouse. B. 3D reconstruction of a whole mount preparation of seminiferous tubule, demonstrating decreasing tomato fluorescence towards the lumen. C. Stra8-Tom mice were produced by mating Stra8-icre males with CAG-LSL-tdTomato females (left). Stra8 icre-RosaYFP males were produced by mating Stra8 icre males with Rosa-LSL-YFP females (right). Shown are frozen sections counterstained with DAPI. Note that while the Rosa locus drives expression throughout the sperm lineage, expression driven by the CAG promoter gradually diminishes. The luminal fluorescence represents autofluorescence of sperm tails. D. DNA content was measured by adding Hoechst to testis cell suspensions followed with FACS analysis based on tdTomato intensity (before sorting) vs. Hoechst. Alternatively, cell populations were sorted as described, ethanol- fixed and stained with propidium iodide. The purity of cell populations based on DNA content after sorting was estimated as following: Undiff.Spg-100% 2C, Diff.Spg, PreL and LZ – mixed population of cells between 2C (incomplete replication) and 4C (complete replication), PDD – mostly 4C cells with slight contamination with 2C and about 14% 1C, Sec.Sp85% 2C and 15% 1C, RS-100% 1C. E. Isolated populations were attached to slides by cytospin centrifugation and immunostained for PLZF and DMRT1 (markers of spermatogonia). 90% of undifferentiated spermatogonia (ckit negative) were strongly positive for both markers, 90% of differentiating spermatogonia (ckit positive) were weakly positive for both markers and absent in all other populations. PLZF expression was 4.4, 4.8 and 10 fold higher in Undiff.Spg versus Diff.Spg in 3 respective experiments. GFR-α (another Undiff.Spg marker) expression was 8.7, 10 and 14-fold higher in Undiff.Spg. F. PreL cells were isolated and nuclear spreads were stained with anti [file pgen.1011785.s001.tif]

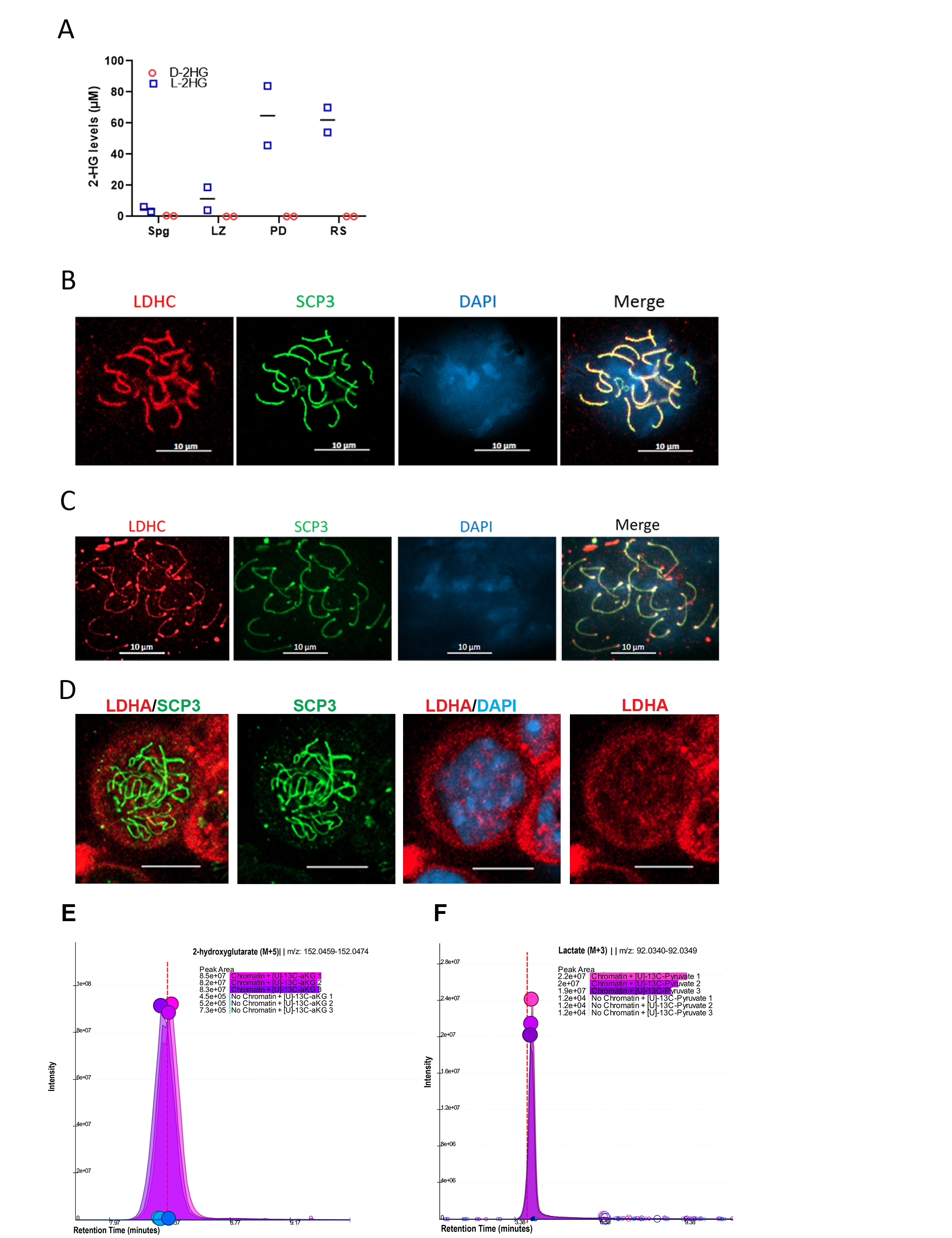

Supplement: S2 Fig — A. L- and D-2HG enantiomer content was measured in the indicated populations using chiral derivatization. Results of two independent experiments are shown. Calculation of 2HG concentrations was performed using the volumes of cells in S2 Table. D-2HG enantiomer was detected only in Spg cells at negligible concentrations of 0.6 and 0.4 μM. B-D: Verification of LDHC staining along chromosomes and in centromeres. B. Freshly isolated testicular cells fixated for 10 sec in -20o methanol were stained with goat Abcam ab3966 LDHC antibody (and not rabbit Proteintech 19981-A) and co-stained with SCP3, compare with Fig 3A. C. Spreads prepared using Papanikos et al. [81], protocol described in methods 29. LDHC was stained with rabbit Proteintech 19981-A and co-stained with SCP3. Strong staining of centromeres with LDHC can be noted. Scale bar 10 µm. D: LDHA is not localized on chromosomes. Confocal microscope image of a diplotene cell stained with LDHA antibody, scale bar 10 µm, compare with Fig 3A. E-F. Catalytic activity of chromatin-bound LDHC was measured as described in the Methods. LC-MS was used to quantify metabolite levels. The M + 5 peak area of L-2HG (E) and the M + 3 peak area of lactate (F) were measured in the presence or absence of chromatin and either U-¹³C-αKG (E) or U-¹³C-pyruvate (F), respectively (n = 3 technical replicates). (TIF) [file pgen.1011785.s002.tif]

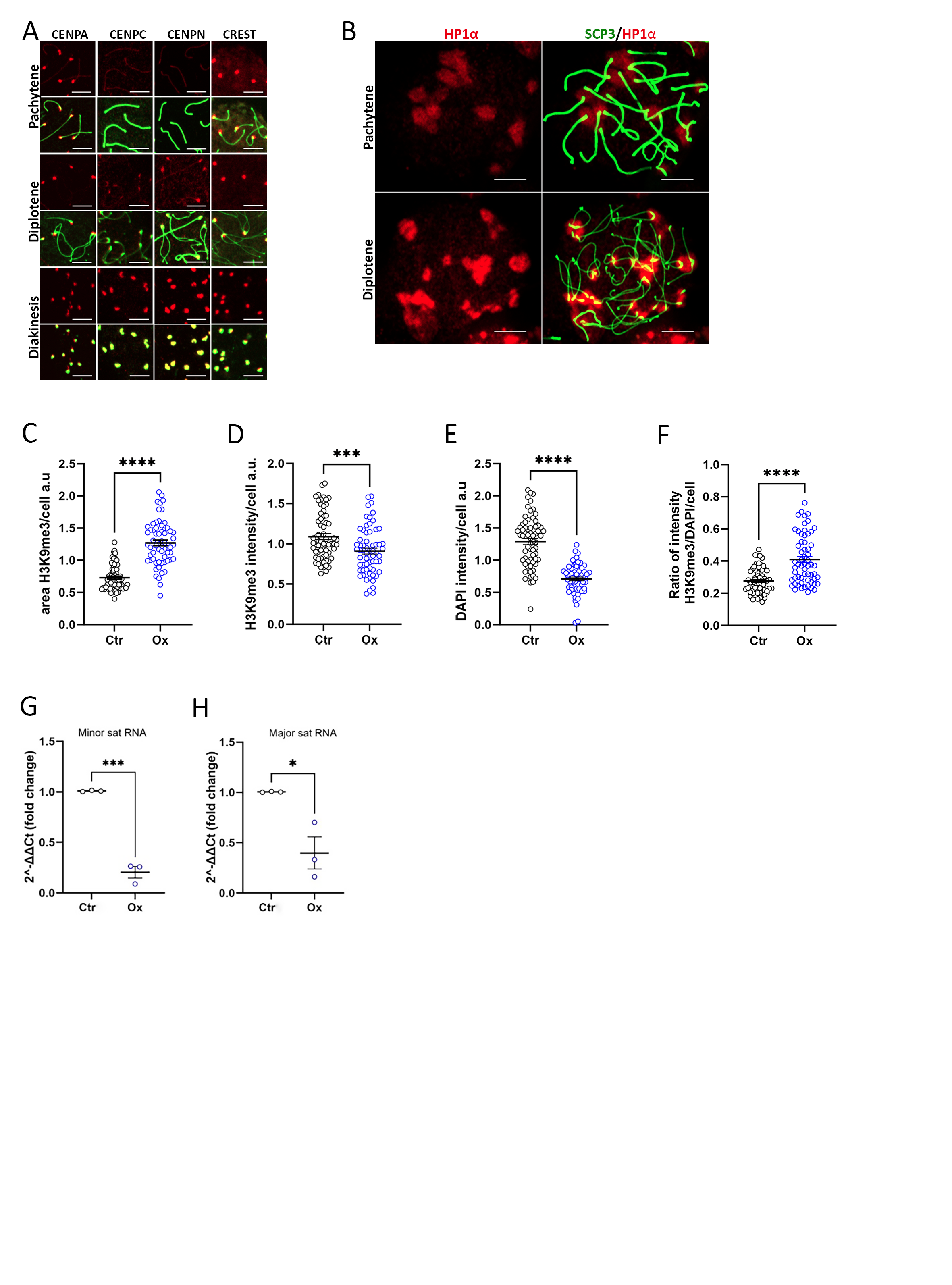

Supplement: S3 Fig — A.Changes in centromeres composition during prophase 1. Pachytene, diplotene and diakinesis cells were stained with SCP3 (green) and CENPA, CENPC, CENPN and CREST antibodies (red). Note gradual appearance of CENPC and CENPN as compared with CENPA. B. Chromocenters demarcated by HP1α antibody are more prominent in diplotene cells as compared to pachytene cells. C-H PDD cells were isolated from two groups: mice injected once with oxamate (1.3g/kg) and sacrificed after 24 hours (Ox) and mice injected with vehicle (Ctr). C-F. Chromocenters of diplotene cells (n = 60, analyzed with a t-test) stained with H3K9me3 were evaluated for the following parameters: area (C), staining intensity (D), DAPI intensity (E), and the ratio of H3K9me3 to DAPI intensities (F). G-H qPCR performed for minor (G) or major (H) satellite RNA. Data represent mean±SE from 3 experiments, t-test. (TIF) [file pgen.1011785.s003.tif]

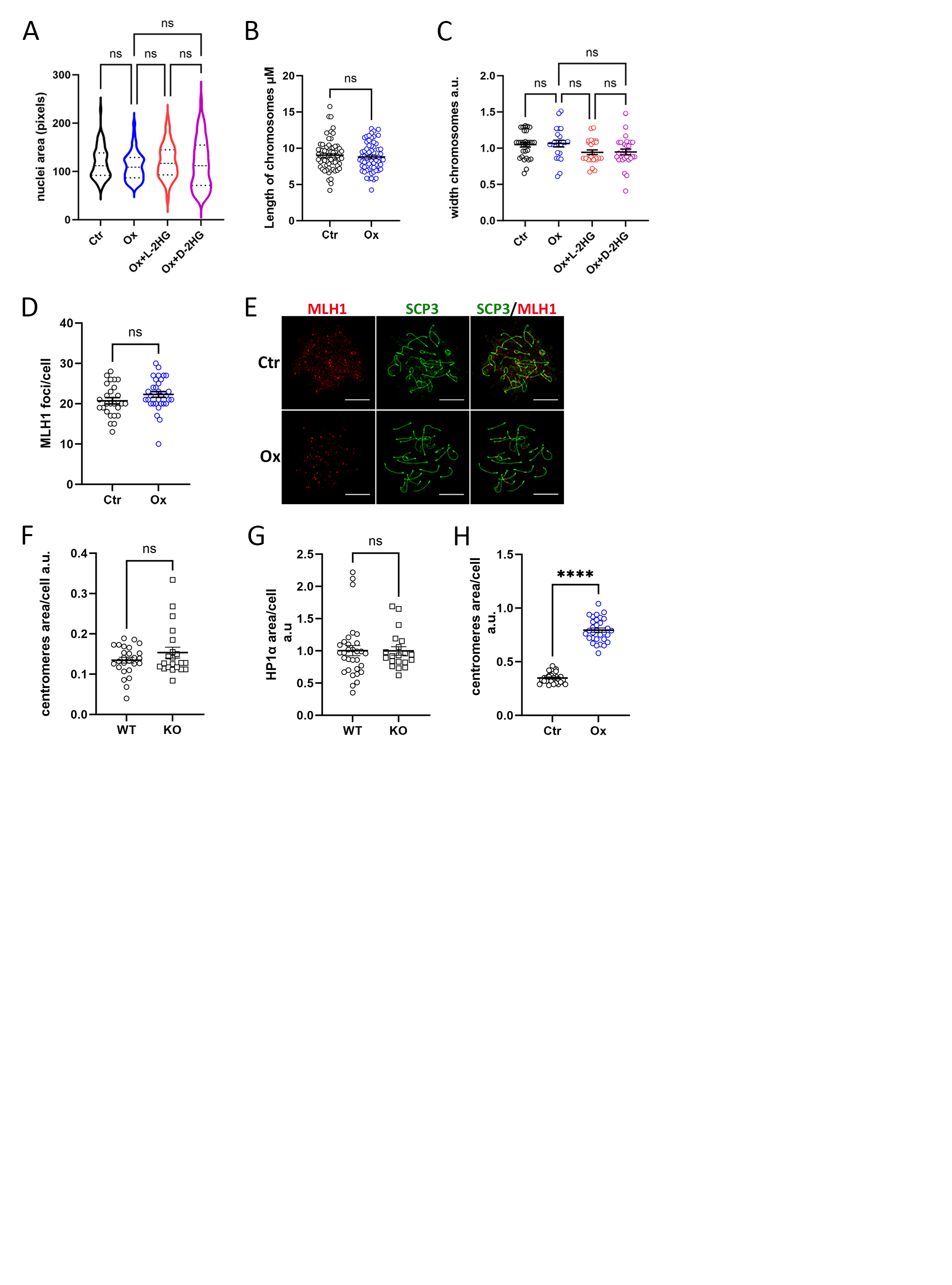

Supplement: S4 Fig — A-C. The PDD population was isolated and cultured in medium with vehicle, 24 mM oxamate, and 24 mM oxamate supplemented with 0.3 mM octyl-L/D 2HG for 24 hours. A. After incubation, cells were attached to slides using cytospin and fixated for 10 seconds in -20°C methanol. SCP3 staining was performed to identify diplotene cells, and nuclei area was measured based on DAPI staining (70–100 nuclei in each group, one-way ANOVA). B-C. Nuclear spreads were prepared after incubation, and pachytene chromosomes were identified using SCP3 staining. B. Chromosome length was measured using ImageJ. Each dot represents the mean length of chromosomes of one cell, with 60 cells per group (Mann-Whitney). C. Width was measured using Zeiss software. Each dot represents the mean width of chromosomes of one cell, with 20–30 cells in each group (one-way ANOVA). D-E and H. PDD cells were isolated from two groups: mice injected once with oxamate (1.3g/kg) and sacrificed after 24 hours (Ox), and mice injected with vehicle (Ctr). D. Cells were stained with SCP3 and MLH1. The number of MLH1 foci was counted in 25 and 30 cells of the respective groups, with mean ± SE calculated (t-test). E. A representative image of MLH1 staining is shown, with a scale bar of 10 µm. F-G. Cells were isolated, and nuclear spreads were prepared from WT and LDHC-KO mice. Cells were stained either with CREST and SCP3 to measure centromere area (F) or with HP1α and SCP3 to measure chromocenter area (G). There were 20–25 diplotene cells in each group (t-test). H. Diplotene cells from control and oxamate-injected mice were stained for CREST and SCP3, and centromere area was measured in 30 cells in each group (t-test). (TIF) [file pgen.1011785.s004.tif]

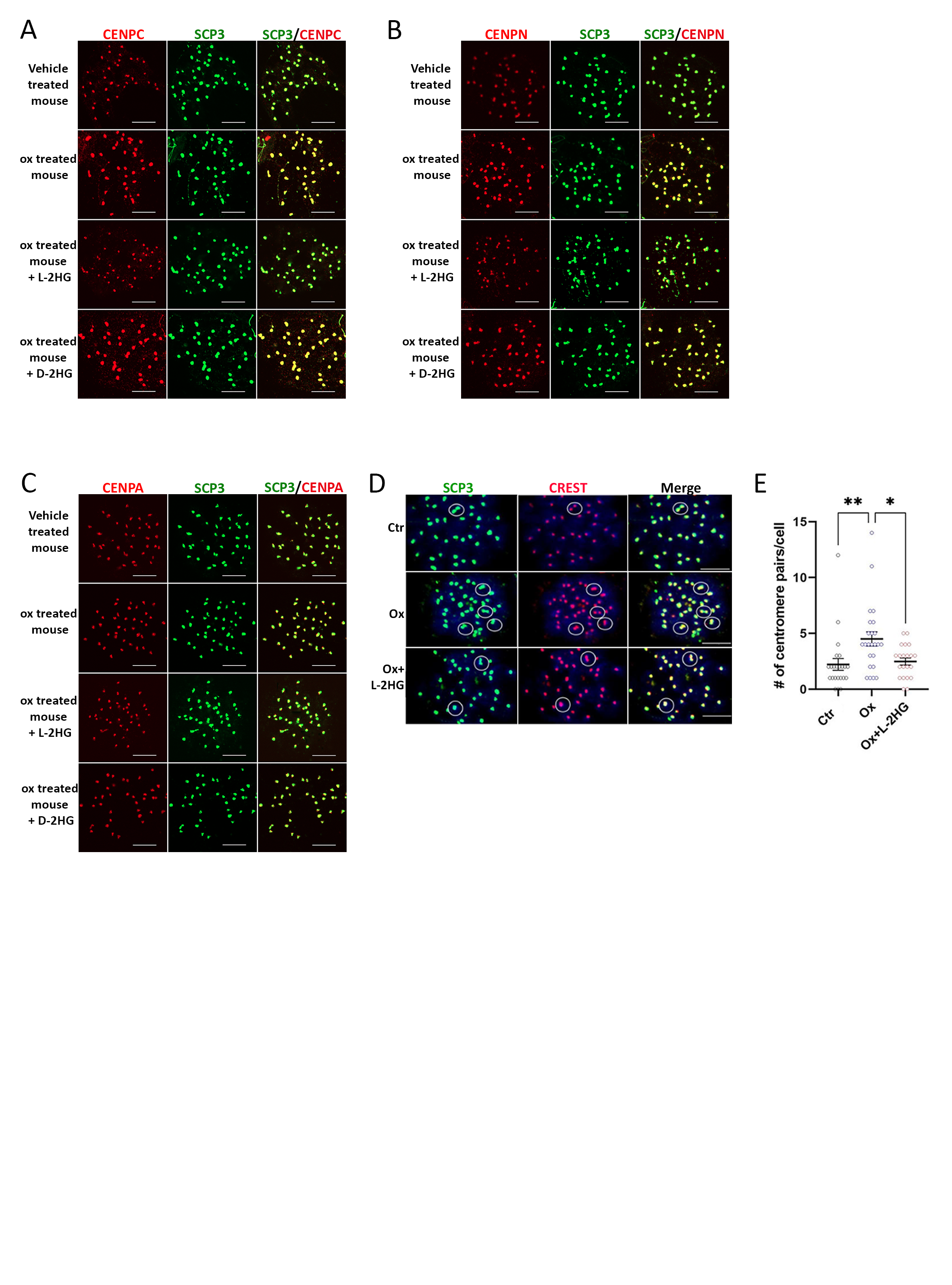

Supplement: S5 Fig — A-B. The PDD population enriched for diakinesis cell content was cultured in medium with vehicle, 24 mM oxamate, and 24 mM oxamate supplemented with 0.3 mM octyl-L-2HG for 24 hours. Following incubation, nuclear spreads were prepared and stained with SCP3 and CREST. A. Representative images with white circles marking paired centromeres are shown, with a scale bar of 10 µm. B. The number of centromere pairs was counted in 20 cells, mean ± SE, one-way ANOVA. (TIF) [file pgen.1011785.s005.tif]

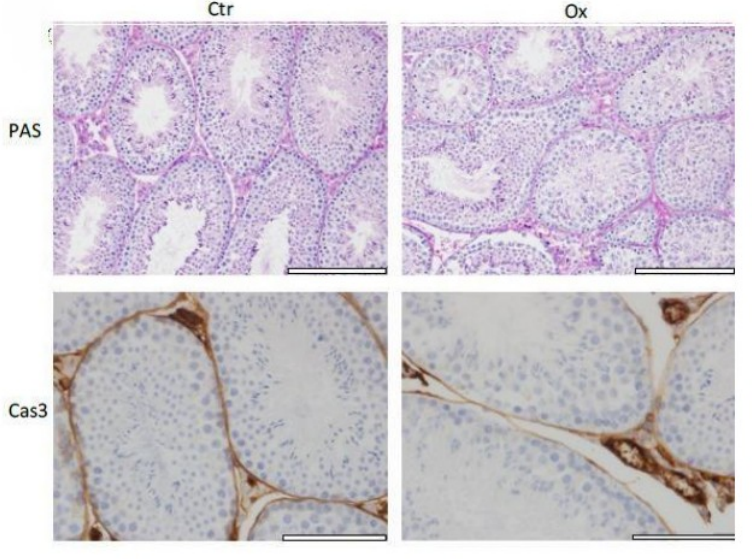

Supplement: S6 Fig — Mice were treated with daily oxamate injections 1.3 g/kg or vehicle for 7 days (n = 6). Paraffin sections were stained with PAS for morphological evaluation or with cleaved caspase 3 for identification of apoptotic cells. Representative images one out of 6 mice, scale bar 100 µm. (TIF) [file pgen.1011785.s006.tif]
