# Supplementary material for: L-2-hydroxyglutarate regulates centromere and heterochromatin conformation in the male germline
Source: PLoS Genet. 2025 Jul 10;21(7):e1011785. doi: 10.1371/journal.pgen.1011785 (PMC12306753; doi:10.1371/journal.pgen.1011785)
Supplement: S1 Table — Gene expression of LZ (6 days in culture) and PDD (48 hours)- normalized reads, mean ±SE of 3 samples. Data from GSE169014. Data were from freshly isolated (e.g., not cultured) cells from GSE162740. (PDF) [file pgen.1011785.s007.pdf]

Supplementary Table 1 Cultured cells are not significantly contaminated with myoid cells.

Gene expression of LZ (6 days in culture) and PDD (48 hours)- normalized reads, mean ±SE of 3 samples. Data from GSE169014. Data of freshly isolated, not cultured cells from GSE162740.

Gene name	Characteristics	LZ-6 days culture	PDD-48 hours culture	LZ-fresh not cultured	PDD-fresh not cultured
PGK1	Chromosome X	333.2±9.4	38.6±2.4		
PDHA1		153.6±16.8	0.0±0.0		
IDH3G		763.0±62.7	18.7±1.2		
Col1A1	Myoid cells markers	28.8±4.9	9.1±0.7	46.8±5.9	2.4±1.8
ACTA2		2.3±0.8	5.3±2.8	1.8±0.9	9.5±3.0
HK1	Glycolysis enzymes	83.2±16.1	14606±874.0		
GAPDH		767.9±30.5	169.3±9.1		
LDHA		116.6±3.6	24629±1035.0		
