# Supplementary material for: L-2-hydroxyglutarate regulates centromere and heterochromatin conformation in the male germline
Source: PLoS Genet. 2025 Jul 10;21(7):e1011785. doi: 10.1371/journal.pgen.1011785 (PMC12306753; doi:10.1371/journal.pgen.1011785)
Supplement: S2 Table — Cell volumes were measured using two independent methods – eclipse and diameter measurement – under the microscope, and there was a high level of agreement in results from the two methods. The volume ratio between the respective cells was calculated based on the average of the two methods. (PDF) [file pgen.1011785.s008.pdf]

	Diameter µm	Radius µm	Surface Area μm^2	Volume μm^3
Eclipse				
Spg	12.4	6.2	486	1008
LZ	9.9	5.0	308	509
PD	16.9	8.4	895	2518
RS	11.3	5.6	398	747
Microscope				
Spg	13.2	6.6	544	1195
LZ	10.6	5.3	351	618
PD	18.0	9.0	1022	3073
RS	12.9	6.5	526	1136
Average of ***Eclipse*** and Microscope				
Spg	12.8	6.4	515	1099
LZ	10.2	5.1	329	562
PD	17.5	8.7	957	2786
RS	12.1	6.1	460	928

Table S2: Cell size parameters of the different cell populations. Cell volumes were measured using two independent methods: eclipse and diameter measurement under the microscope, with high level of agreement between the two methods. The volume ratio between the respective cells was calculated based on the average of the two methods.
