# Supplementary material for: L-2-hydroxyglutarate regulates centromere and heterochromatin conformation in the male germline
Source: PLoS Genet. 2025 Jul 10;21(7):e1011785. doi: 10.1371/journal.pgen.1011785 (PMC12306753; doi:10.1371/journal.pgen.1011785)
Supplement: S3 Table — Genes from clusters 3 and 9 of the PDD population and clusters 1 and 2 of the RS population were analyzed using the metadatabase GeneAnalytics software, with the pathway enrichment significance threshold set at FDR < 0.05. (PDF) [file pgen.1011785.s009.pdf]

	PDD cluster 3 and 9	RS cluster 1 and 2
JmjC histone demethylation	KDM2B	KDM2B
		KDM3A
	KDM4A	KDM4A
	KDM5B	KDM5B
	KDM5C	KDM5C
		KDM6B
Histone demethylation		ARIDSB
Histone methylation		NSD3
		KMT2B
		CARM1
		SETD1A
Histone acetylation	NCOA1	EP300
	MSL1	
	MSL3	
	YeatS2	
	KAT7	
	CREBBP	CREBBP
		HAT1
		TAF5L
		BRD1
		RUVBL1
Histone deacetylation	ZZZ3	
	GATAD2A	GATAD2A
		GATAD2B
	Sirt1	
		UHRF1
Histone modifications/remodelling	MORF4C2	
	HCFC1	
	BM1	
		BRWD1
		ARID1A
		PBRM1
		SMARCC1

Table 3: Summary of significant effects attributed to alterations in L-2HG levels on gene categories related to chromatin organization and remodelling.

Genes from cluster 3 and 9 of the PDD population and cluster 1 and 2 of the RS population were analyzed using the metadatabase GeneAnalytics software, with the pathway enrichment significance threshold set at FDR < 0.05.
