# Supplementary material for: L-2-hydroxyglutarate regulates centromere and heterochromatin conformation in the male germline
Source: PLoS Genet. 2025 Jul 10;21(7):e1011785. doi: 10.1371/journal.pgen.1011785 (PMC12306753; doi:10.1371/journal.pgen.1011785)
Supplement: S4 Table — (DOCX) [file pgen.1011785.s010.docx]

**S4 Table: List of Antibodies Used in the Study**

| **antibody** | **Cat #** |
| --- | --- |
| Caspase 3 | 9661 (Cell Signaling) |
| CD117 (cKit- APC conjugated) | 105812 (Biolegend) |
| CENP-A | C51A7 (Cell Signaling) |
| CENP-C | NBP2-75438 (Novus) |
| CENP-N | 16751-1-AP ((Proteintech) |
| Crest | 15235 (Antibody INC) |
| DMRT1 | SC-377167 (Santa Cruz) |
| H3K9me3 | ab8898 (Abcam) |
| HP1α | AB-ab109028 (Abcam) |
| LDHA | 199871-A (Proteintech) |
| LDHC | 199891-A (Proteintech) |
| LDHC | ab3966 (Abcam) |
| PhosphoH3 (serine10) | 36916 (Cell Marque) |
| PhosphoH3 (serine 10) | MA5-15220 (Invitrogen) |
| PLZF | AB5535 (Millipore) |
| SCP3 | ab15093 (Abcam) |
| SCP3 | ab97672 (Abcam) |
| SOX9 | AB5535 (Millipore) |
| γH2AX | 05-636 (Millipore) |
| MAD1 | 18322-1-AP (Proteintech) |
| MLH1 | 551092 (BD pharmingen) |
| STAR ORANGE | 00206JR-3 (Abberior) |
| STAR RED | 00121JR-1 (Abberior) |
| Cy5 donkey anti rabbit | ab150075 (Abcam) |
| Alexa488 goat anti mouse | Ab150117(Abcam) |
| Cy5 donkey anti human | 709175 149 (Jackson) |
