# Supplementary material for: L-2-hydroxyglutarate regulates centromere and heterochromatin conformation in the male germline
Source: PLoS Genet. 2025 Jul 10;21(7):e1011785. doi: 10.1371/journal.pgen.1011785 (PMC12306753; doi:10.1371/journal.pgen.1011785)
Supplement: Attachment — Submitted filename: Response to reviewers.docx [file pgen.1011785.s014.docx]

**Response to reviewers**

**We thank the reviewers for their comments and have revised our manuscript extensively to address their comments.**

**Specifically, we have performed additional experiment to test the putative enzymatic activity of lactic dehydrogenase C (testis specific) bound to chromatin of meiotic cells at the stage of pachytene/diplotene , as requested by the reviewer 1.**

**Additionally, we have substantially edited our introduction and discussion sections as requested by reviewers 2 and 3. We believe that these changes have considerably enhanced our manuscript. Our detailed point-by-point responses to the reviewers’ comments are in bold below.**

**Changes in the revised manuscript are highlighted as follows: yellow for revisions requested by Reviewer #1, blue for those requested by Reviewer #2, and green for those requested by Reviewer #3.**

Reviewer #1: This study identifies L-2HG as a critical regulator of centromere and heterochromatin organization in spermatogenesis, revealing a novel metabolic-chromatin link. It provides strong evidence that nuclear LDHC associates with meiotic chromosomes and plays a key enzymatic role. The finding that L-2HG depletion disrupts pericentric heterochromatin and centromere integrity is convincing. The proposed role of polymer-polymer phase separation (PPPS) in centromere structure adds mechanistic depth, with potential implications for male fertility and genome stability. The study is well-executed, well-written, and conceptually innovative. However, several concerns need to be addressed:

1. How does L-2HG regulate chromosome dynamics during meiosis? One possible mechanism is its direct binding to chromatin modifiers (Fig. 9). Additionally, the authors show in Fig. 7 that L-2HG manipulation induces global transcriptomic changes in genes controlling chromatin modifications, suggesting transcriptional regulation. Please clarify this point.

**This point was clarified in the Discussion section (line 682 to 697).**

2. While Fig. 3 demonstrates nuclear localization of LDHC, it remains unclear whether nuclear LDHC has a metabolic function in L-2HG production. Additional experiments are needed to differentiate the roles of cytoplasmic and nuclear LDHC.

**To test whether chromatin bound LDHC is catalytically active, we set out to compare the enzymatic activity of nuclear and cytoplasmic LDHC. To this end, we isolated chromatin from pachytene/diplotene/diakinesis (PDD) cells following the same protocol used for examining chromatin-bound proteins, with the exception that the protein extraction step was omitted. We assessed LDHC activity on chromatin using 13C-labeled α-ketoglutarate or 13C-pyruvate as substrates, to detect the formation of 13C-L-2HG and 13C-L-lactate, respectively. This procedure is described in detail in the newly added 'Methods' section (Lines 1107–1124). We observed significant LDHC activity associated with chromatin, as reported in the 'Results' section (Lines 262–268), and we discuss the potential implications of this finding in the 'Discussion' (Lines 644–650).**

3. The use of oxamate to block L-2HG synthesis lacks specificity. Although this concern is partially mitigated by the rescue experiment using octyl-L-2HG, siRNA-mediated LDHC knockdown is feasible with the current culture system and would provide more direct evidence.

**The reviewer proposed verifying our results with oxamate by using siRNA to acutely decrease LDHC expression. While theoretically sound, this experiment is not feasible, as pachytene/diplotene cells are extremely fragile, survive only 48 hours in culture, and do not tolerate transduction-based manipulations. Despite employing several different methodologies over the years we've studied this system, we have consistently been unable to transduce cells within this primary culture system.**

- Reviewer #2: This study by Mayorek and colleagues examines the noncanonical functions of Lactate Dehydrogenase C (LDHC) in the mouse testis. Previous studies from Schatz and Segal in 1969 described how LDHC (then called LDHX) from mouse testis can synthesize 2HG from a-ketoglutarate. This function was rediscovered and elaborated on by the Rabinowitz group in 2016 – at which point the question was raised as to what endogenous function this LDHC-L2HG axis served in the mouse testis. Here the authors conduct a series of well-controlled experiments that reveal an important function for LDHC in centromere condensation and heterochromatin organization.

  The manuscript is divided into two sections. The first section describes a novel genetic tool that enables sorting of testis cell-types. While this section could be better integrated with the latter 2/3 of the manuscript, the methodology appears sound and will likely be of broader interest. The second portion of the manuscript focuses on the function of LDHC on producing L-2HG in a subset of testis cell types, with a majority of efforts directed on the role of LDHC and L-2HG in regulating: (i) LDHC localization to chromatin, and (ii) maintaining centromeric heterochromatin. The experiments are well-controlled and convincing. Overall, the authors demonstrate that L-2HG promotes LDHC localization to chromatin. Moreover, their findings indicate that loss of L-2HG production disrupts heterochromatin density.

  I think this is a landmark study – for too long, studies of both D-2HG and L-2HG have focused on disease phenotypes. Nearly all manuscripts focus on the links between the two 2HG enantiomers and either oncogenic mutations or human disease phenotypes. But an ever increasing body of evidence indicates that both D-2HG and L-2HG have important endogenous functions – bacteria generate D- and L-2HG, yeast generate D-2HG, Diptera – the second-largest order of animals on planet earth generate millimolar quantities of L-2HG during juvenile growth. Yet, studies of D- and L-2HG almost always focus on cancer metabolism.

  My major criticism for the manuscript is that I'd encourage the authors to break out of the biomedical troupe that D- and L-2HG are oncometabolites and instead embrace the true importance of their findings. Theirs is the first conclusive evidence that L-2HG serves an important endogenous role during animal development. I’d strongly suggest that the authors restructure their introduction and discussion to claim this primacy. While they should feel free to discuss the oncogenic roles of these molecules, I’d note that they have the opportunity to restructure the manuscript in a manner that shifts the entire field towards thinking about the normal roles of the 2HGs. There’s plenty of evidence that these molecules play essential roles in development – you have the most concrete evidence to date.

**The changes were introduced in the "Introduction", lines 88-95 and in the "Discussion" section: lines 749-756 in line with the above very important comment.**
I’d also insist that the authors clean up aspects of the manuscript related to L-2HG metabolism. Throughout the manuscript, the authors mention 2HG as if it’s one molecule. Be specific wherever possible – use L-2HG instead of 2HG unless the assay was ambiguous. Similarly, the authors conduct a series of elegant rescue experiments using octyl-L2HG. There are several instances where the authors note adding D- or L-2HG to cells – be specific, if these experiments involved octyl-2HG, please note this appropriately.

**All corrections concerning the identification of 2HG enantiomers were introduced according to the above comment.**

A few other notes for the text:

The intro mentions production of D-2HG in mammals due to oncogenic mutations in IDH. D-2HG is also produced under endogenous conditions due to the enzyme HOT and from serine biosynthesis.

**Corrected in the "Introduction" section: lines 74-75 with new relevant references #9 and 10**

The results sections focused on the new transgenic tools are conceptually divorced from the remainder of the manuscript. The authors should consider some edits to this text to setup the remainder of the results section.

**We edited the text according to this comment: lines 109-110 and 173-174.**

Schatz and Segal described how LDHC from testis produces L-2HG in 1969. Sorry if I missed the reference, but this discovery should be given precedence over the Rabinowitz lab discovery in 2016.

**The study of Schatz and Segal was added to our references: # 13**

The 13C labeling experiment, as written, implies that L-2HG is produced directly from lactate. I think the authors mean to imply that lactate is converted to pyruvate and fed into the TCA cycle to generate aKG, which is then used to synthesize L-2HG. Please state so explicitly.

**The detailed explanation was introduced in the "Results" section lines 194-198.**

The finding that oxamate, but not LDHC mutations, exhibit differences in centromere and heterochromatin compaction to be very interesting. The authors try to explain way this difference, but an alternative model is that LDHC unbound by L-2HG is important for the phenotype, and in the LDHC mutants, the absence L-2HG causes LDHC to promote changes in heterochomatin. The authors should raise this possibility.

**We have checked LDHC-KO mice, which lack both the enzyme and L-2HG**.

Please cite appropriate references outside of the oncometabolite literature. The Schatz and Segal study listed above is essential.

**Done, as mentioned above, reference #13.**

In addition, a PNAS study of L-2HG in Drosophila noted that loss of L-2HG results in enhanced position effect variegation phenotypes in the fly eye. This phenotype is known to result from spreading of pericentric heterochromatin and clearly supports the authors findings. While the fly study doesn’t describe a mechanistic basis of this phenomenon – the authors study provides an explanation for this finding in the fly.

**Thank you very much for this insightful comment. We have incorporated the findings in Drosophila into the 'Discussion' section (Lines 749–756) and added the relevant new references (#23 and #80).**

Reviewer #3: This study investigated 2-HG in mouse spermatogenesis. Using biochemical approaches, they showed that 2-HG is most abundant in PDD cells (pachytene diplotene diakinesis spermatocytes) and round spermatids (RS). They further showed that 2-HG is produced by LDHC, a testis-specific LDH. LDHC localizes to the meiotic chromosomal axis and centromeres in spermatocytes. Acute treatment of PDD cells with oxamate, a pan-LDH inhibitor, causes enlargement of centromeres and chromosome centers in PDD cells in culture. Mice treated with oxamate exhibited the same defects in centromeres and chromocenters in spermatocytes. However, LDHC-deficient mice did not show similar defects. Treatment of round spermatids with oxamate also caused increase in chromocenter size. Transcriptomic profiling revealed increased minor and major satellite RNAs upon oxamate treatment. This study supports that conclusion that 2-HG modulates centromere and chromocenter conformation in male germ cells, specifically in meiotic spermatocytes and round spermatids.

This study is well done. The manuscript is well-written. Discussions are balanced. Alternative explanations (redundant enzymatic functions etc) were provided. I support its publication after addressing the following concerns.

Major concerns:

1) The title needs to be revised: “2-hydroxyglutarate controls centromere and heterochromatin conformation and function in the male germline”. This study didn’t examine the function of centromere and heterochromatin. Therefore, “function” needs to be deleted in the title. “control” is too strong. Suggested title: 2-hydroxyglutarate regulates centromere and heterochromatin conformation in the male germline.

**We have revised the title in accordance with the reviewer's suggestion.**

2) Testis is naturally hypoxic. PDD cells and round spermatids are behind the blood-testis barrier and reside in a hypoxic environment. Could the high 2-HG in the testis help germ cells to adapt to hypoxia? A brief discussion would be useful to readers.

**We appreciate this insightful comment. We have expanded on the issue of the hypoxic environment in the seminiferous tubules in the 'Discussion' section (Lines 651–661) and added references #68, #69, and #70.**

3) Localization of LDHC to the chromosomal axis (synaptonemal complex) and centromeres in PDD cells. This localization pattern of LDHC in PDD cells (meiotic prophase I cells) is novel and very interesting. However, the function of this localization remains unknown, because the LDHC-deficient males produced sperm and lacked meiotic defects. At least it is not required for meiotic progression. LDHC knockout males were infertile due to failure for the knockout sperm to fertilize oocytes.
4) Lines 103-104: This statement needs to be deleted: “These effects are necessary for the proper progression of meiosis and thus directly link 2HG to normal meiotic progression”. The data supporting this statement were weak, because the mice were only studied at 7 days. This might not be long enough to see the chromosome segregation defects. No meiotic defects were shown except for the MAD1 signal. In addition, activation of SAC would lead to apoptosis in spermatocytes. Increased apoptosis was not shown.

**As requested, we deleted the original statement and replaced it with a more carefully worded sentence: 'These findings may suggest a direct link between L-2HG and normal meiotic progression' (Lines 103–104).**
